# Revealing prognostic and tumor microenvironment characteristics of cuproptosis in bladder cancer by genomic analysis

**DOI:** 10.3389/fgene.2022.997573

**Published:** 2022-10-03

**Authors:** Shun Zhang, Shenggen Yu, Huangqi Duan, Weimin Xia, Chen Wang, Haibo Shen

**Affiliations:** Department of Urology, Xinhua Hospital, School of Medicine, Shanghai Jiao Tong University, Shanghai, China

**Keywords:** bladder cancer, cuproptosis, prognosis, tumor microenvironment, risk score

## Abstract

**Objectives:** Bladder cancer (BLCA) is the most common malignant tumor in the urinary system, while the prognosis of muscle-invasive bladder cancer (MIBC) is poor. Cuproptosis might be a promising therapeutic approach to trigger tumor cell death. This study aimed to figure out the role of cuproptosis in BLCA and constructed a new cuproptosis scoring system to guide clinical diagnosis and individualize treatments.

**Methods:** Consensus clustering was used to classify 490 patients with BLCA from TCGA and GEO cohorts. Survival outcomes and functional enrichment analyses were performed between the different subtypes. The cuproptosis scoring system was constructed by LASSO-Cox analysis. ESTIMATE, CIBERSORT, and ssGSEA were used to investigate the tumor microenvironment (TME). Drug sensitivity was evaluated with pRRophetic. An immunotherapy cohort was used to investigate the treatment response. The cuproptosis scoring system was verified in our own cohort with quantitative real-time PCR.

**Results:** An overview of 12 cuproptosis genes (CuGs) in the TCGA database was depicted. Based on the mRNA expression profiles of CuGs, patients were classified into two cuproptosis molecular patterns. Based on the differential genes between the two cuproptosis patterns, the patients were classified into two cuproptosis gene clusters. There were distinct survival outcomes, signaling pathways, and TME between the two subtypes. A 7-gene cuproptosis scoring system was constructed. Patients with high cuproptosis scores showed worse OS and more immunosuppressing TME than those with low cuproptosis scores. The two cuproptosis score groups had distinct mutation profiles. Patients with high cuproptosis scores tended to be sensitive to chemotherapy drugs, but insensitive to immune checkpoint inhibitors (ICIs) treatment.

**Conclusion:** This study depicted the landscape of cuproptosis in BLCA. We constructed a cuproptosis scoring system to predict the prognosis of BLCA patients. There were significant differences in survival outcomes, TME, mutation profiles, and drug sensitivities in high and low cuproptosis score patients. The cuproptosis scoring system could help oncologists comprehensively understand the tumor characteristic of BLCA and make individualized treatment strategies.

## Introduction

Bladder cancer (BLCA) is the most common malignant tumor in the urinary system, with 573,278 new cases and 212,536 new deaths in 2020 worldwide (Sung et al., 2021). According to the statistics, there will be about 81,180 estimated new cases and 17,100 estimated deaths of both sexes in the United States in 2022 (Siegel et al., 2022). For over 4 decades, adjuvant cisplatin-based combination chemotherapy after radical surgery (RC) remains the primary curative treatment choice for muscle-invasive bladder cancer (MIBC) (Witjes et al., 2021), however, the 5-year survival of regional and distant metastatic BLCA is only 28–39% and 5–6% respectively (Siegel et al., 2022). Based on the results of some clinical trials, immune checkpoint inhibitors (ICIs) were investigated in advanced bladder cancer. Currently, 5 ICIs (atezolizumab, pembrolizumab, nivolumab, durvalumab, and avelumab) are approved by the US Food and Drug Administration (FDA) for the treatment of patients with advanced BLCA (Lobo et al., 2017). In addition, understanding of molecular profiling of BLCA helped to develop targeted therapies, such as fibroblast growth factor receptor (FGFR) inhibitors. Erdafitinib, a pan-FGFR inhibitor, is the most extensively studied and is currently the only FDA-approved FGFR inhibitor to treat advanced BLCA (Patel et al., 2020). However, only a small portion of patients can benefit from immunotherapy and targeted therapy. Existing prognostic predictive markers like Tumor-Node-Metastasis (TNM) stage and biomarkers like programmed cell death-Ligand 1 (PD-L1) expression level did not perform as well as expected (Powles et al., 2018). So, it is urgent to find out a new predictive system to guide clinical diagnosis and individualize treatments.

Copper is an essential cofactor for all organisms, but it becomes toxic if concentrations exceed a threshold. Tsvetkov et al. found that copper binds to lipoylated components of the tricarboxylic acid (TCA) cycle, resulting in proteotoxic stress and ultimately leading to a novel form of cell death termed cuproptosis (Tsvetkov et al., 2022). The researchers performed a whole-genome CRISPR–Cas9 screen and identified several key genes involved in copper-induced cell death, including the ferredoxin1 (FDX1), lipoyl synthase (LIAS), lipolytransferase 1 (LIPT1), dihydrolipoamide dehydrogenase (DLD), dihydrolipoamide S-acetyltransferase (DLAT), pyruvate dehydrogenase E1 subunit alpha 1 (PDHA1), pyruvate dehydrogenase E1 subunit beta (PDHB), ATPase copper transporting alpha (ATP7A), ATPase copper transporting beta (ATP7B), solute carrier family 31 member 1 (SLC31A1), and dihydrolipoamide branched chain transacylase E2 (DBT). The researcher observed that cells undergoing mitochondrial respiration are particularly sensitive to copper ionophores. Furthermore, FDX1 and lipoylated proteins are highly correlated across a diversity of human tumors, suggesting that cuproptosis could play an important role in tumors with such a metabolic profile and induction of cuproptosis might be a promising therapeutic approach to trigger tumor cell death. However, the expression profile of the little-known cuproptosis-related genes in tumors and their association with patients’ prognosis remains unknown. The tumor characteristics, tumor microenvironment (TME), and drug sensitivity of patients with different cuproptosis genes (CuGs) expression levels are still elusive.

In the present study, we depicted an overview of the CuGs in BLCA patients in the TCGA database. Then cuproptosis molecular patterns and cuproptosis gene clusters with distinct survival and TME features were identified. We further constructed a cuproptosis risk score system to predict survival for each BLCA patient and evaluated the tumor characteristics, TME features, and drug sensitivity of the patient with different cuproptosis scores. The scoring system may give oncologists guidance for prognosis prediction and clinical treatments.

## Methods

### Datasets

The RNA-seq and clinicopathological data were downloaded from The Cancer Genome Atlas database (TCGA, https://portal.gdc.cancer.gov) up to 20 April 2022, and the Gene Expression Omnibus database (GEO, https://www.ncbi.nlm.nih.gov/geo). The external validation cohort to evaluate the response of ICI was from the IMvigor210 study, a cohort of platinum-treated locally advanced or metastatic urothelial carcinoma (mUC) patients receiving anti-PD-L1 immunotherapy. The gene expression profiles were normalized using the “limma” R package. After excluding those without complete clinical data, a total of 490 patients in the TCGA and GEO cohorts and 298 patients in the IMvigor210 cohort were included in this study. The clinicopathological characteristics of the samples were provided in Supplementary Data S1. CNV and somatic mutation data were obtained from the TCGA database.

### Differential analysis

The differentially expressed genes (DEGs) between tumor tissues and para-carcinoma tissues or between two cuproptosis patterns was identified by using the “limma” R package with a false discovery rate (FDR) < 0.05 in the cohorts and |logFC| > 1.

### Consensus clustering analysis

Consensus clustering was applied to identify distinct cuproptosis-related molecular patterns based on the expression of cuproptosis genes and cuproptosis-related gene clusters based on differential genes between the two cuproptosis patterns. The number of unsupervised clusters, and their stability, were determined by the consensus clustering algorithm using the “ConsensuClusterPlus” package (Wilkerson and Hayes, 2010). PCA was applied to verify the subtype assignments.

### Function analysis

Gene set variation analysis (GSVA) was performed in heatmap by using “GSVA” R package. “c2.cp.kegg.v7.4.symbols” was chosen as reference. An adjusted *p* < 0.05 was considered to be significantly enriched. Gene ontology (GO) and Kyoto Encyclopedia of Genes and Genomes (KEGG) were conducted using the “clusterProfiler” R package.

### Cuproptosis score model

LASSO algorithm was applied to construct cuproptosis score models with the “glmnet” R package. Risk scores of the patients were calculated according to the normalized expression level of each gene and its corresponding regression coefficient. Then the patients were divided into the high-risk group and low-risk group based on the median values of the risk score. The receiver operating characteristic (ROC) curve was used to evaluate the predictive power of our models using the “survival”, “survminer”, and “timeROC” R packages. “Survival” package was used to perform the univariate and multivariate Cox regression analyses. Based on the risk score and different clinical features (gender, age, T stage), a nomogram model was established to predict the 1-,3-, and 5-years survival for the patients using the “rms” and “survival” packages (Fu and Song, 2021).

### Estimation of the tumor microenvironment

Single sample gene set enrichment analysis (ssGSEA) was used to evaluate the infiltrated levels of 16 immune cell subtypes between the two groups with “gsva” R package (Yoshihara et al., 2013). Immune checkpoints were extracted from previous studies and their expressions between the two groups were compared by Wilcoxon test (Morad et al., 2021). The CIBERSORT algorithm (https://cibersort.stanford.edu/) was used to estimate the correlation of the relative abundances of distinct immune cell types and risk scores based on gene expression in tumor tissues (Gentles et al., 2015).

### Estimate

Stromal and immune scores of each sample were generated by ESTIMATE algorithm (Yoshihara et al., 2013). The ESTIMATE score was calculated based on the stromal and immune scores, which was negatively correlated with tumor purity.

### Molecular classifier, tumor neoantigen burden, microsatellite instability score, and tumor mutation burden

Molecular subtypes and TNB data of the TCGA dataset were extracted from supplementary data of a previous study (Robertson et al., 2017). MSI score was obtained from the TCGA database. TMB of the TCGA dataset was obtained from UCSC Xena (http://xena.ucsc.edu/) and calculated by (total count of variants)/(the whole lengths of exons).

### Drug sensitivity evaluation

The sensitivity (relative IC_50_) of each patient to chemotherapy drugs was estimated by the “pRRophetic” R package based on Cancer Genome Project (CGP) data (Geeleher et al., 2014). The response of each patient to ICI was evaluated in the IMVigor210 cohort.

### RNA extraction and quantitative real-time PCR

Total RNA was extracted from patients’ tumor samples using TRIzol (ThermoFisher, 15596026), then reversely transcribed to cDNA by using PrimeScript RT Reagent Kit (TaKaRa, RR014A). Quantitative real-time PCR (qPCR) was performed using Universal Blue SYBR Green qPCR Master Mix (Servicebio, G3326-15). The 2^-△CT^ method was used for data analyses. Primers for qRT-PCR were synthesized by Biosune (Shanghai) and were shown in Supplementary Table S1.

### Statistical analysis

Statistical analysis was conducted using R (version 4.0.3). Comparisons between two groups were performed using Wilcoxon rank-sum test. Kaplan- Meier curves were used for OS analysis by log-rank test. Correlation coefficients were computed by Spearman’s distance correlation analyses. All statistical p values were two-sided and *p* < 0.05 was considered statistically significant.

### Patients and specimens

A total of ten pairs of normal and bladder cancer tumor samples and 20 tumor tissues were collected from patients in Xinhua hospital affiliated to Shanghai Jiao Tong university school of medicine. The detailed clinicopathological characteristics of the patients were in Supplementary Data S2.

## Results

### An overview of cuproptosis genes in the TCGA database

**FIGURE 1:**
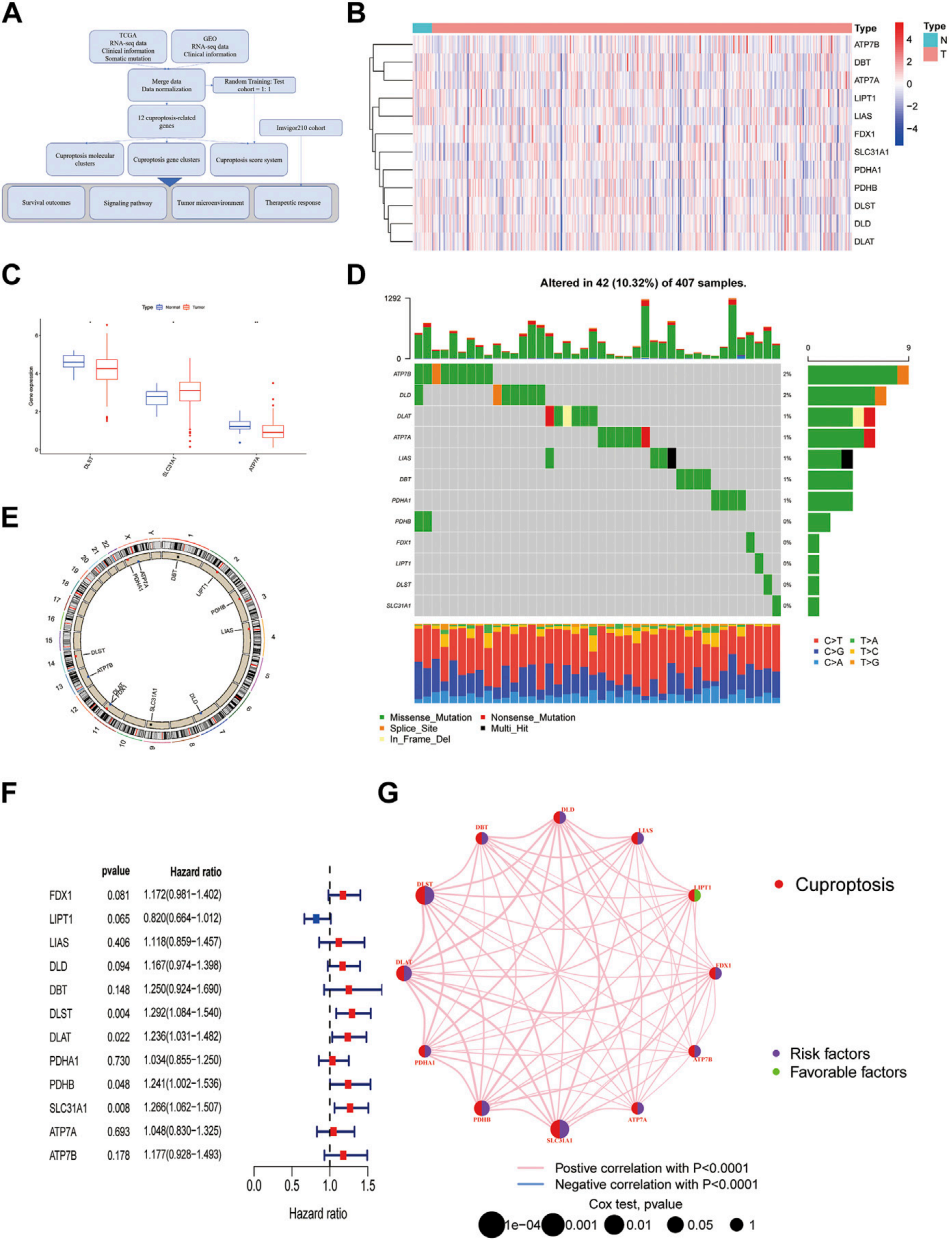
An Overview of Cuproptosis genes in the TCGA database. **(A)** The diagram of the study. **(B)** Heatmap of 12 CuGs expressions among normal and tumor samples in the TCGA cohort. **(C)** Differential CuGs among normal and tumor samples in the TCGA cohort. **(D)** The landscape of mutation profiles of the CuGs in 407 patients from the TCGA cohort. **(E)** The CNV frequency and location of the CuGs on chromosomes. **(F)** Forest plots showing the results of the univariate Cox regression analysis of CuGs that were correlated with OS. **(G)** Expression correlation network of the CuGs. Positive correlations were shown in red lines. Negative correlations were shown in blue lines. The risk factors were shown in purple circles, while the favorable factors were shown in green circles. *p < 0.05, **p < 0.01. Abbreviations: TCGA, The cancer genome atlas; CuGs, cuproptosis genes; CNV, copy number variation; OS, overall survival.

The diagram of this study was shown in Figure 1A. For clustering and developing a scoring system, BLCA patients from the TCGA cohort and the GEO cohort (GSE31684) were enrolled. Detailed clinical data could be found in Supplementary Data S1. 12 cuproptosis-related genes (CuGs) were chosen according to the previous studies (Tang et al., 2022). Figure 1B showed their distribution among normal and tumor samples in the TCGA cohort. DLST and ATP7A had a higher expression in normal tissues, while SLC31A1 had a higher expression in tumor tissues (Figure 1C). 42 of the 407 samples (about 10.32%) showed cuproptosis-related gene mutations. Of these, ATP7B showed the highest frequency of mutations. Most of the mutations were missense mutations (Figure 1D). The copy number variation (CNV) frequency of these CuGs and their locations on chromosomes were shown in Figure 1E. 4 of the CuGs (DLST, DLAT, PDHB, SLC31A1) were associated with overall survival (OS) by the univariate Cox regression analysis (*p* < 0.05) (Figure 1F). Figure 1G showed that the CuGs all had positive expression correlation. Except for LIPT1, all the CuGs were risk factors of OS in BLCA.

### Cuproptosis molecular patterns with distinct survival and TME features in BLCA

**FIGURE 2:**
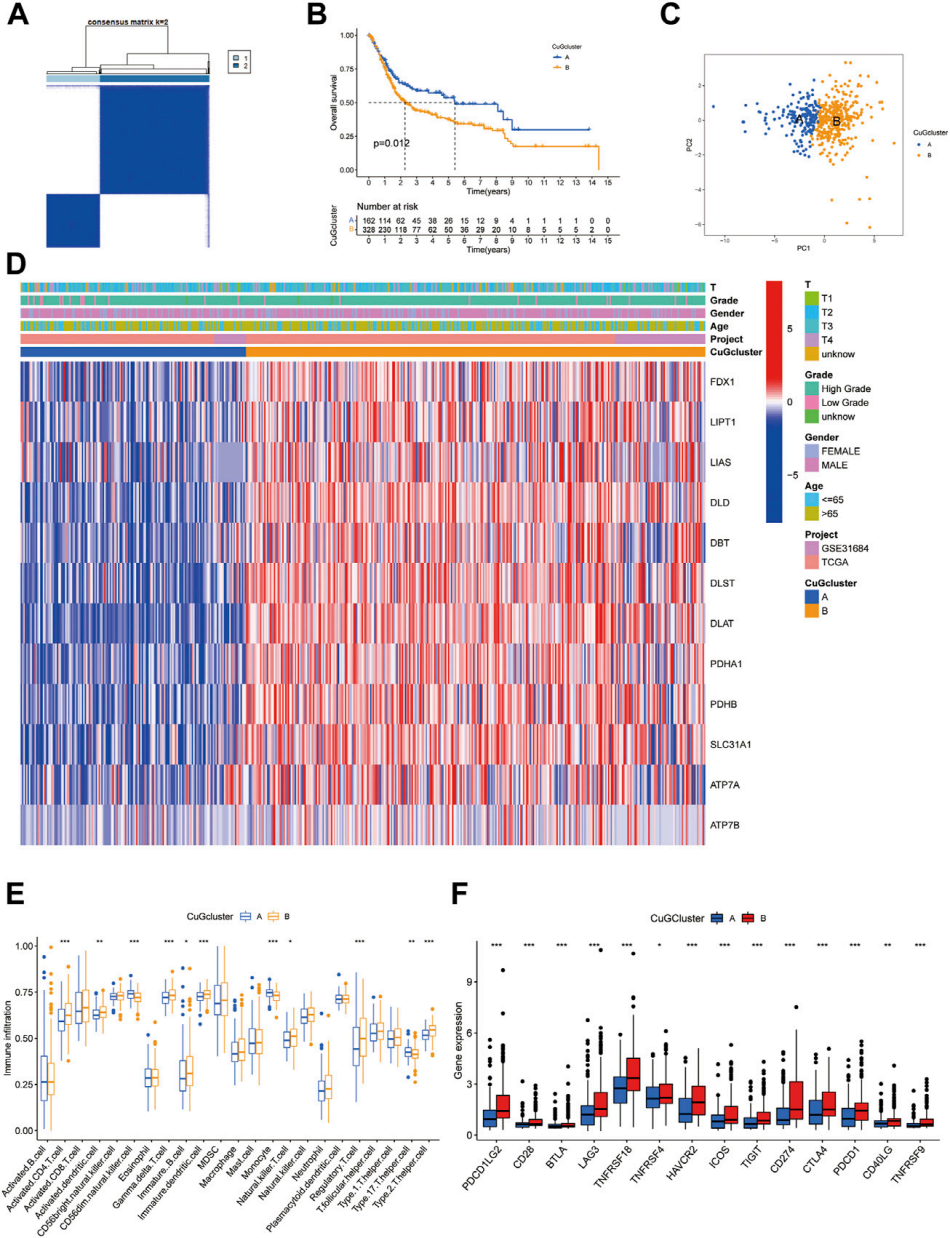
Cuproptosis-related molecular patterns with distinct survival and TME features. **(A)** The consensus score matrix of all the samples by unsupervised clustering analysis based on the mRNA expression profiles of 12 CuGs. **(B)** Kaplan-Meier curves for the two cuproptosis molecular patterns. **(C)** PCA of the two cuproptosis molecular patterns: pattern A (blue) and pattern B (orange). **(D)** Heatmap showed the clinical features and expression of CuGs of the two patterns. **(E)** Boxplot showed different immune cell infiltration between the two patterns by ssGSEA analysis. **(F)** Boxplot showed different immune checkpoints expression between the two patterns. *p < 0.05, **p < 0.01, ***p < 0.001. Abbreviations: TME, tumor microenvironment; CuGs, cuproptosis genes; PCA, principal component analysis; ssGSEA, single sample gene set enrichment analysis.

Based on the mRNA expression profiles of 12 CuGs in TCGA and GSE31684, patients were classified into two molecular patterns (A: n = 162, B: n = 328) by unsupervised clustering analysis (Figure 2A, Supplementary Figure S1, Supplementary Data S3). Patients in cuproptosis pattern A had a significant better overall survival than those in pattern B (Figure 2B). Principal component analysis (PCA) enabled us to visualize that the patient in different molecular patterns could be distinguished well (Figure 2C). Different clinical features and expression of CuGs of the two patterns were shown in Figure 2D. Patients with longer survival time in pattern A had lower expression of CuGs, confirming that most CuGs were risk factors of OS in BLCA. The correlation between the molecular patterns and tumor immune microenvironment was also evaluated. ssGSEA analysis showed significant difference in immune cell infiltration between the two patterns. Natural killer cell (NK), monocytes, Type 17 T helper cell (Th17) showed higher infiltration in pattern A. However, immune-suppressing cells like immature dendritic cell (DC), regulatory T cell (Treg) and type 2 T helper cell (Th2) showed higher abundance in pattern B, which might contribute to the poor outcome of patients in pattern B (Figure 2E). To further explore the immune statuses, we compared the expression of the immune checkpoints between the two patterns. Surprisingly, all the immune checkpoints including CD274 (also named PD-L1), Programmed Cell Death Protein 1 Ligand 2 (PDCD1LG2, also named PD-L2), Programmed Cell Death Protein 1 (PDCD1, also named PD1), Cytotoxic T lymphocyte antigen-4 (CTLA4), T cell immunoglobulin and mucin domain-3 protein (Tim-3, also named HAVCR2), Lymphocyte-activation gene 3 (LAG-3), T cell immunoglobulin and ITIM domain (TIGIT), CD28, Inducible T cell costimulatory (ICOS), B- and T-lymphocyte attenuator (BTLA), TNF receptor super-family member 18 (TNFRSF18, also named GITR), TNF receptor super-family member 4 (TNFRSF4, also named OX40), TNF receptor super-family member 9 (TNFRSF9, also named 4-1BB), CD40 ligand (CD40LG) showed higher expression in pattern B (Figure 2F).

### Enrichment analysis of differential genes between cuproptosis-related molecular patterns

**FIGURE 3:**
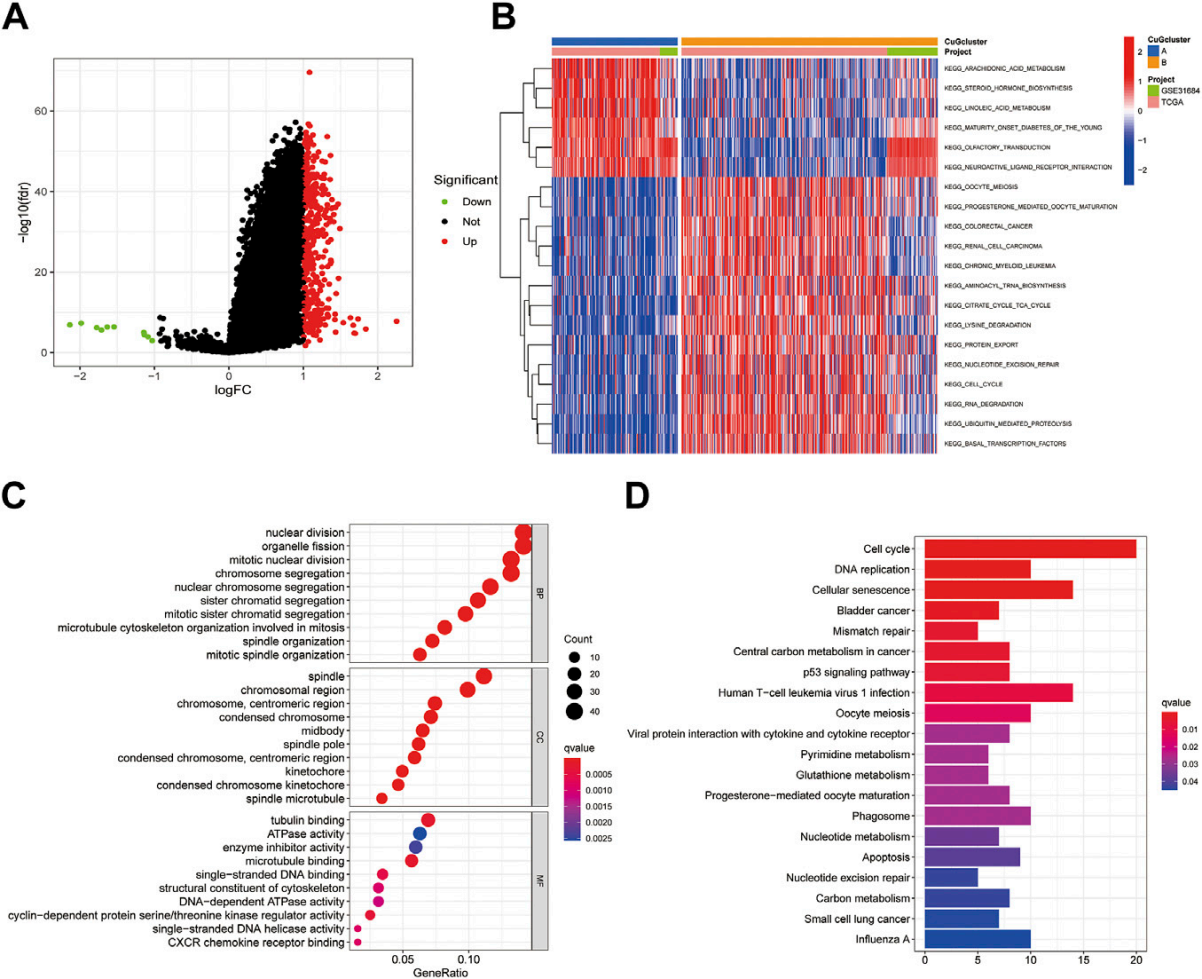
Enrichment analysis of differential genes between cuproptosis-related molecular patterns. **(A)** Volcano plot showed the differential genes between cuproptosis-related molecular patterns. Up-regulated genes in pattern B were shown in red and down-regulated genes were shown in green. Genes of no significance were shown in black. **(B)** GSVA enrichment analysis of the differential genes. **(C)** GO enrichment analysis of BP, CC, and MF results ranked by gene ratio. **(D)** KEGG pathway analysis of the differential genes. Abbreviations: GSVA, gene set variation analysis; GO, gene ontology; BP, biological process; CC, cellular component; MF, molecular function; KEGG, kyoto encyclopedia of genes and genomes.

To further understand the biological behaviors between the two cuproptosis patterns, differential expression analysis was conducted. 10 down-regulated genes and 369 up-regulated genes were identified (|logFC| >1, fdr <0.05) (Figure 3A). Then the differential genes were sent to gene set variation analysis (GSVA) enrichment analysis (Figure 3B). Pattern A showed higher activities of lipid biosynthesis and metabolism, like arachidonic acid metabolism, linoleic acid metabolism, and steroid hormone biosynthesis. While pattern B showed higher activities on the TCA cycle, Lysine degradation, and cell cycle. Consistently, GO and KEGG analysis also showed enrichment in nuclear division and cell cycle, which might be a possible target for patients who had high expression of CuGs (Figures 3C,D).

### Prognostic and TME characteristics between two cuproptosis gene clusters in BLCA

**FIGURE 4:**
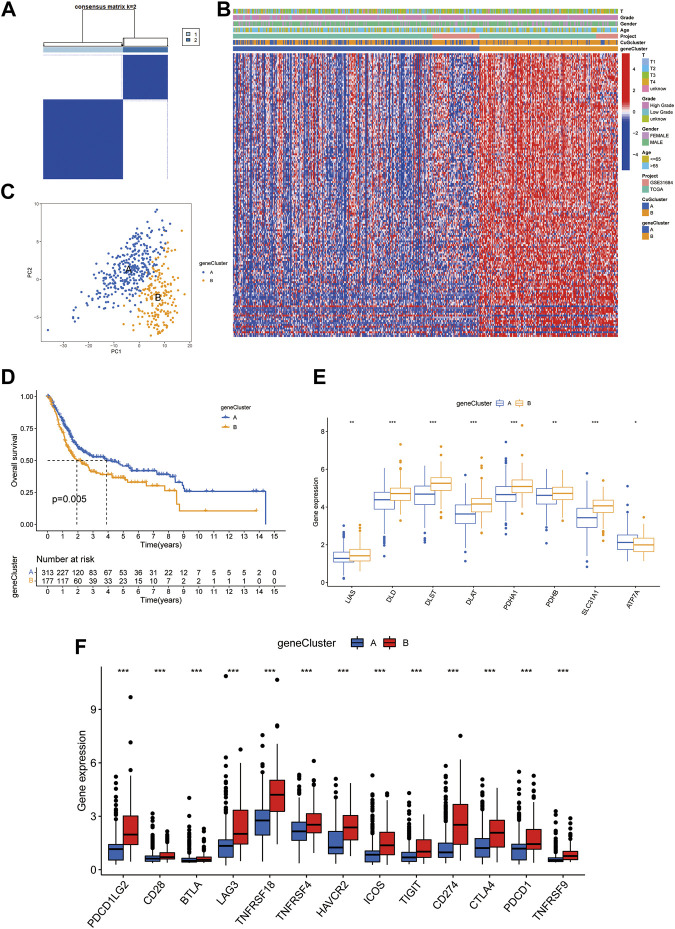
Prognostic and TME characteristics between two cuproptosis gene clusters in BLCA. **(A)** The consensus score matrix of all the samples by unsupervised clustering analysis based on the differential genes between the two cuproptosis patterns. **(B)** Heatmap depicted the clinical features and expression of differential genes between the two gene clusters. **(C)** PCA of the two gene clusters: cluster A (blue) and cluster B (orange). **(D)** Kaplan-Meier curves for the two gene clusters. **(E,F)** Boxplots showed different CuGs **(E)** and immune checkpoints **(F)** expression between the two gene clusters. *p < 0.05, **p < 0.01, ***p < 0.001. Abbreviations: TME, tumor microenvironment; BLCA, bladder cancer; PCA, principal component analysis; CuGs, cuproptosis genes.

Based on the differential genes between the two cuproptosis patterns, unsupervised clustering was performed, and the patients were newly classified into two gene clusters (A: n = 313 B: n = 177) (Figure 4A, Supplementary Figure S2, Supplementary Data S4). The heatmap visualized the expression of the differential genes between two gene clusters and two cuproptosis patterns (Figure 4B). PCA confirmed that the two gene clusters could be completely distinguished (Figure 4C). Patients in cluster A had significant longer overall survival time than those in cluster B (Figure 4D). Figure 4E exhibited the expression of CuGs between the two gene clusters. Most CuGs were expressed lower in cluster A. To further assess the TME difference between the two gene clusters, the expression of immune checkpoints was also analyzed. In cluster B, all the immune checkpoints showed higher expression, indicating a more immuno-suppressing TME (Figure 4F).

### Generation of the cuproptosis scoring system to predict survival of BLCA patients

**FIGURE 5:**
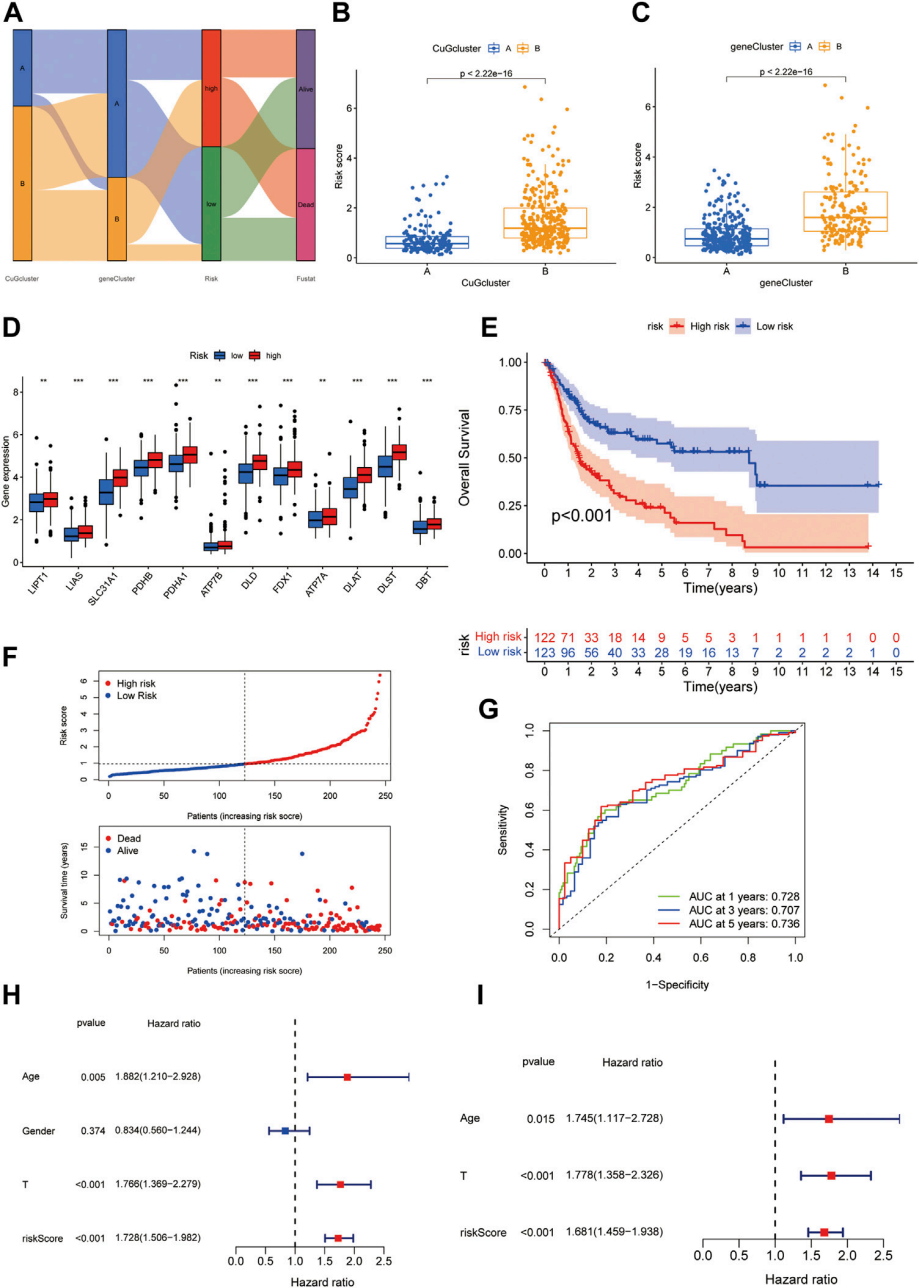
Generation of the cuproptosis scoring system to predict survival of BLCA patients. **(A)** Alluvial diagram of two cuproptosis patterns, two gene clusters, cuproptosis scores, and survival status. **(B,C)** Boxplot depicted the differences in cuproptosis scores between the cuproptosis patterns **(B)** and gene clusters **(C)**. **(D)** Boxplot showed the different CuGs expression in the two risk groups. **(E)** Kaplan-Meier survival analysis for patients in high- and low-risk groups in the training cohort. **(F)** The distribution of the risk scores, OS statues, and the correlations between OS and risk scores. **(G)** ROC curve of the cuproptosis risk scoring system for prediction of OS in the training cohort. **(H)** Results of the univariate Cox regression analyses regarding OS in the training cohort. The risk score was significantly associated with the OS. **(I)** Results of the multivariate Cox regression analyses regarding OS in the training cohort. The risk score was an independent prognostic factor. *p < 0.05, **p < 0.01, ***p < 0.001. Abbreviations: CuG, cuproptosis genes; OS, overall survival; ROC, receiver operating characteristic.

To better apply these subtypes to clinical outcome prediction and treatments, we established a prognostic model to calculate a specific score for every patient. Univariate Cox regression and multi-variate cox regression analysis identified 7 differential genes associated with OS between the two cuproptosis patterns. Then the patients were randomly assigned to two groups, i.e., the training group (n = 245) and the validation group (n = 245) (Supplementary Data S5). LASSO Cox regression analysis was applied to establish the prognostic model using the expression profile of the 7 genes. The cuproptosis risk score was calculated by the following formula for each patient: 0.199 * expression level of PRMT5 + 0.147 * expression level of CNN3 + 0.143 * expression level of TM4SF1 + 0.099 * expression level of DSC3 + 0.082 * expression level of ALDH1A1 - 0.161 *expression level of CXCL11–0.065 * expression level of HMGCS2. Then the patients in the training group were divided into a high-risk group (n = 122) or a low-risk group (n = 123) according to the median cut-off value of the cuproptosis risk score. Figure 5A showed the interaction of cuproptosis score and the survival outcomes in the cuproptosis patterns and the gene clusters. Patients in cuproptosis pattern B and gene cluster B had higher cuproptosis scores (Figures 5B,C). In the high cuproptosis risk group, CuGs showed higher expression (Figure 5D). The Kaplan-Meier curve indicated that patients in the high-risk group had significantly worse overall survival (Figure 5E), which was consistent with the CuGs expression and the former molecular cluster and gene cluster results. The higher cuproptosis score was associated with the poor outcome (Figure 5F). The area under the curve (AUC) of the prognostic model was 0.728 at 1 year, 0.707 at 3 years, and 0.736 at 5 years, suggesting that the cuproptosis risk score had a reliable capacity for predicting the prognosis of BLCA patients (Figure 5G). In univariate Cox regression analyses, the cuproptosis score was significantly associated with OS (HR = 1.728, 95% CI = 1.506–1.982, *p* < 0.001) (Figure 5H). After adjusting for other confounding factors, the cuproptosis score was confirmed to be an independent predictor for OS in multivariate Cox regression analyses (adjusted HR = 1.681, 95% CI = 1.459–1.938, *p* < 0.001) (Figure 5I).

### Validation of the cuproptosis scoring system for BLCA

**FIGURE 6:**
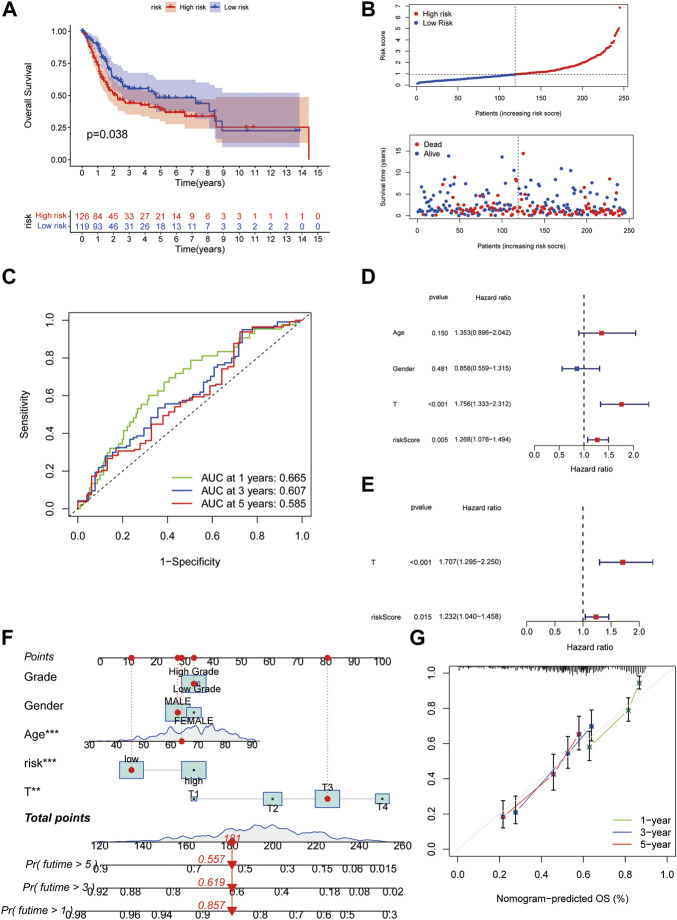
Validation of the cuproptosis scoring system for BLCA. **(A)** Kaplan-Meier survival analysis for patients in high- and low-risk groups in the validation cohort. **(B)**The distribution of the risk scores, OS statues, and the correlations between OS and risk scores. **(C)** ROC curve of the cuproptosis risk scoring system for prediction of OS in the validation cohort. **(D)** Results of the univariate Cox regression analyses regarding OS in the validation cohort. The risk score was significantly associated with the OS. **(E)** Results of the multivariate Cox regression analyses regarding OS in the validation cohort. The risk score was an independent prognostic factor. **(F)** Nomogram for the prediction of 1-,3-, 5-years survival probability in patients with BLCA. The red line showed the score of one patient as an example. **(G)** Calibration curves of nomograms in terms of the agreement between predicted and observed 1-, 3-, and 5- years OS. Abbreviations: OS, overall survival; ROC, receiver operating characteristic; BLCA, bladder cancer.

To validate the cuproptosis scoring system, the same strategy was applied in the validation cohort. The Kaplan-Meier curve also indicated a worse OS in the high-risk group (Figures 6A,B). The AUC at 1-, 3-, 5- years were 0.665, 0.607, and 0.585, suggesting the reliability of the model (Figure 6C). In univariate Cox regression analyses, the risk score was significantly associated with OS (HR = 1.268, 95% CI = 1.076–1.494, *p* = 0.005) (Figure 6D). The risk score was still proved to be an independent predictor for OS in multivariate Cox regression analyses (adjusted HR = 1.232, 95% CI = 1.040–1.458, *p* = 0.015) (Figure 6E). After combined with the other three indexes, a novel nomogram was constructed. For a specific bladder cancer patient in clinical practice, the 1-, 3-, and 5-years survival probability could be predicted based on his/her age, gender, tumor grade, and risk score (Figure 6F). As shown in Figure 6G, the nomogram-predicted OS was very close to the observed OS, suggesting the accuracy of the nomogram. AUC of the nomogram at 1-, 3-, 5- years was 0.756, 0.746, and 0.745 in all the patients, which was better than only using the T stage (AUC at 1-, 3-, 5- years was 0.653, 0.670, 0.672) (Supplementary Figure S3).

### High cuproptosis score was associated with immunosuppressing TME in BLCA

**FIGURE 7:**
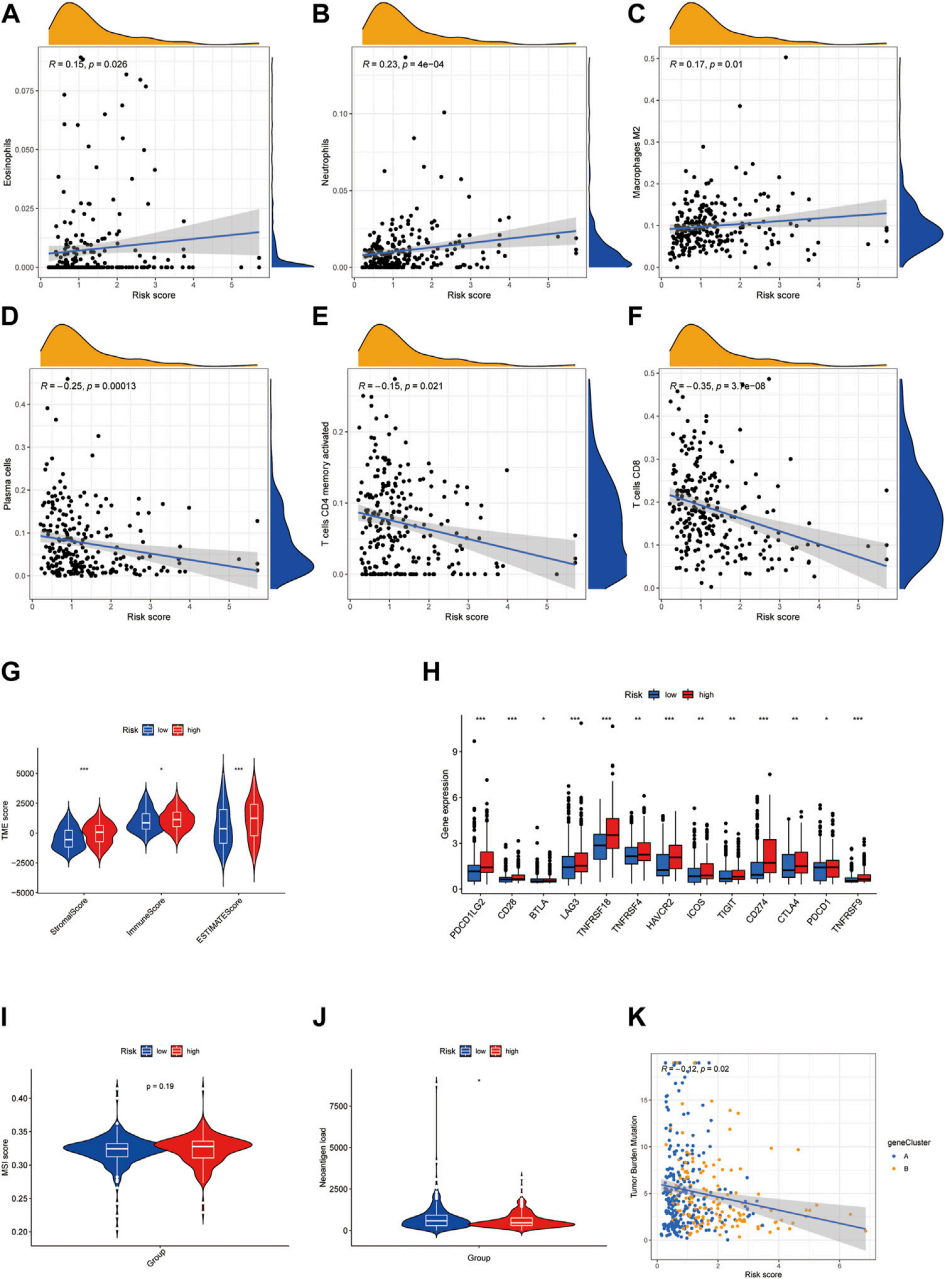
High cuproptosis score is associated with immunosuppressing TME in BLCA. **(A–F)** Correlation of risk score with immune cell infiltration analyzed by CYBERSORT. **(G)** Violin plots showed the difference in stromal score, immune score, and ESTIMATE score in high- and low-risk groups. **(H)** Boxplots exhibited the expression of the immune checkpoint in high- and low-risk groups. **(I,J)** Violin plots showed the MSI score **(I)** and neoantigen load **(J)** in high- and low-risk groups. **(K)** Correlation of risk score with tumor mutation burden. *p < 0.05, **p < 0.01, ***p < 0.001. Abbreviations: TME, tumor microenvironment; BLCA, bladder cancer.

We further probed into the TME and other tumor characteristics of the patients in high and low-risk groups to find out what caused the poor outcome of patients with high cuproptosis scores. Using CYBERSORT analysis, we found that cuproptosis score was positively correlated with eosinophils, neutrophils, and macrophage M2, while it was negatively correlated with plasma cells, activated CD4 memory T cells, and CD8 cells (Figures 7A–F). Furthermore, ESTIMATE was used to analyze the abundance of immune cells and stromal cells. Patients in the high cuproptosis score group had distinct higher stromal (*p* < 0.001) and immune scores (*p* < 0.05) than those in the low cuproptosis score group. The ESTIMATE score was significantly higher in the high-risk group (*p* < 0.001), which suggested a lower tumor purity in the high-risk group (Figure 7G). Immune checkpoints showed higher expression in the high-risk group (Figure 7H). MSI scores showed no difference between the two groups (Figure 7I). However, the high-risk group had lower neoantigen load (Figure 7J), and patients with high-risk scores had lower tumor mutation burden (Figure 7I). Taken together, the high-risk group had a more immunosuppressing TME.

### Different mutation profiles between cuproptosis risk groups

**FIGURE 8:**
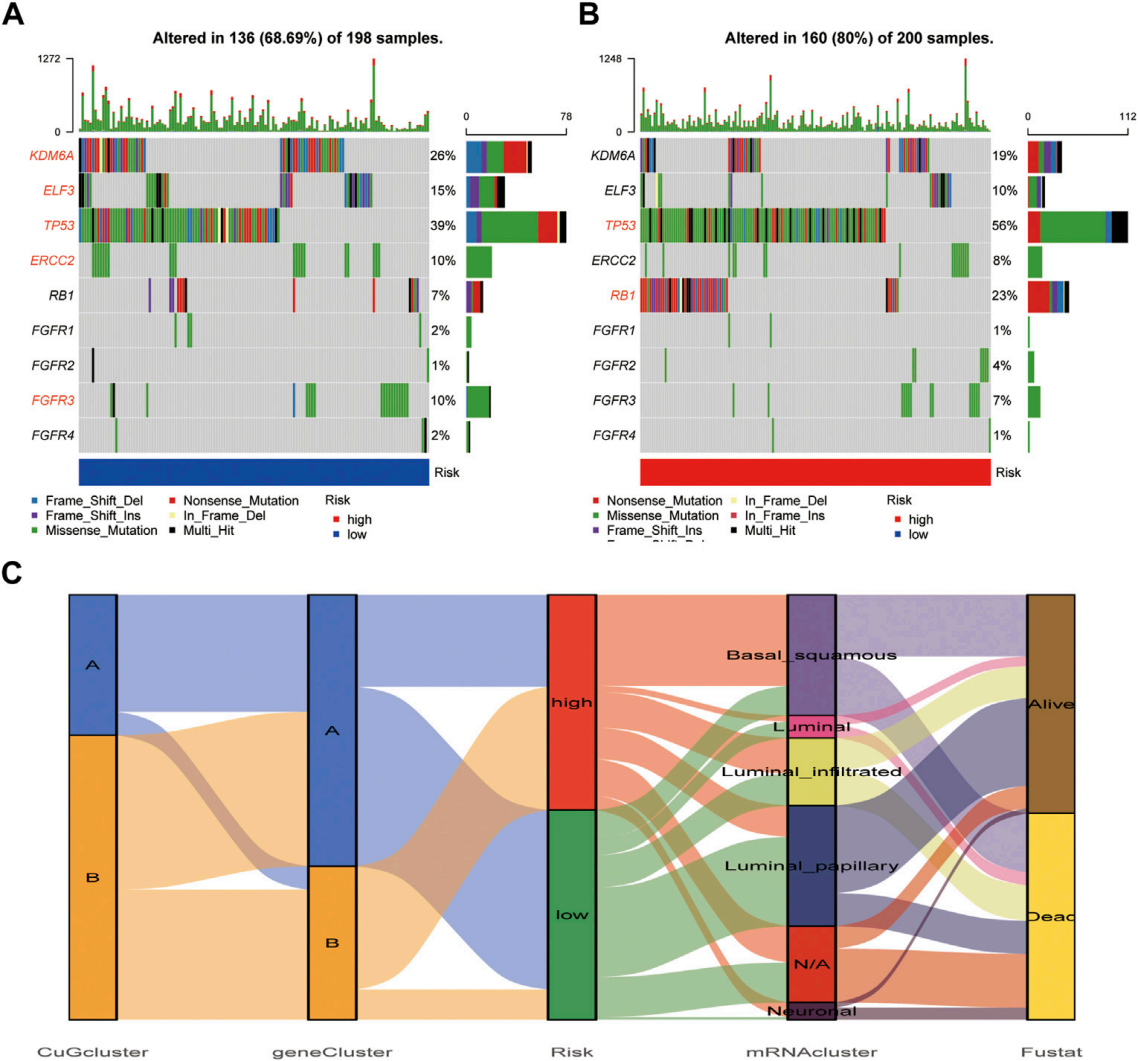
Mutation difference between cuproptosis risk groups. **(A,B)** The waterfall plot showed the mutations of most-concerned genes in BLCA according to the previous research in the low-risk group **(A)** and high-risk group **(B)**. The characteristic genes in each group were emphasized in red. **(C)** The alluvial diagram showed the relationship of cuproptosis risk groups and 2017 TCGA clustering. Abbreviations: BLCA, bladder cancer; TCGA, The cancer genome atlas.

The waterfall plot showed the mutations of most-concerned genes in BLCA according to the previous research between the high-risk group and low-risk group (Figures 8A,B). Mutations of these genes are closely related to the tumor character and final outcome. In the low-risk group, KDM6A, ELF3, TP53, ERCC2, and FGFR3 showed higher mutation frequency, which was more like the luminal, luminal infiltrated, and luminal papillary subtypes according to the 2017 TCGA clustering, which had a relatively better outcome. While in the high-risk group, TP53 and RB1 showed higher mutation frequency, which was more similar to the basal squamous subtype, which had higher immune-checkpoints expression but poor immune response (Robertson et al., 2017). The risk score distribution of TCGA patients in five TCGA subtypes was shown in Figure 8C. The consistency of the cuproptosis risk groups with the TCGA subtypes proved the reliability of the cuproptosis scoring system.

### The roles of the cuproptosis scoring system on response to chemotherapy, targeted therapy, and immunotherapy

**FIGURE 9:**
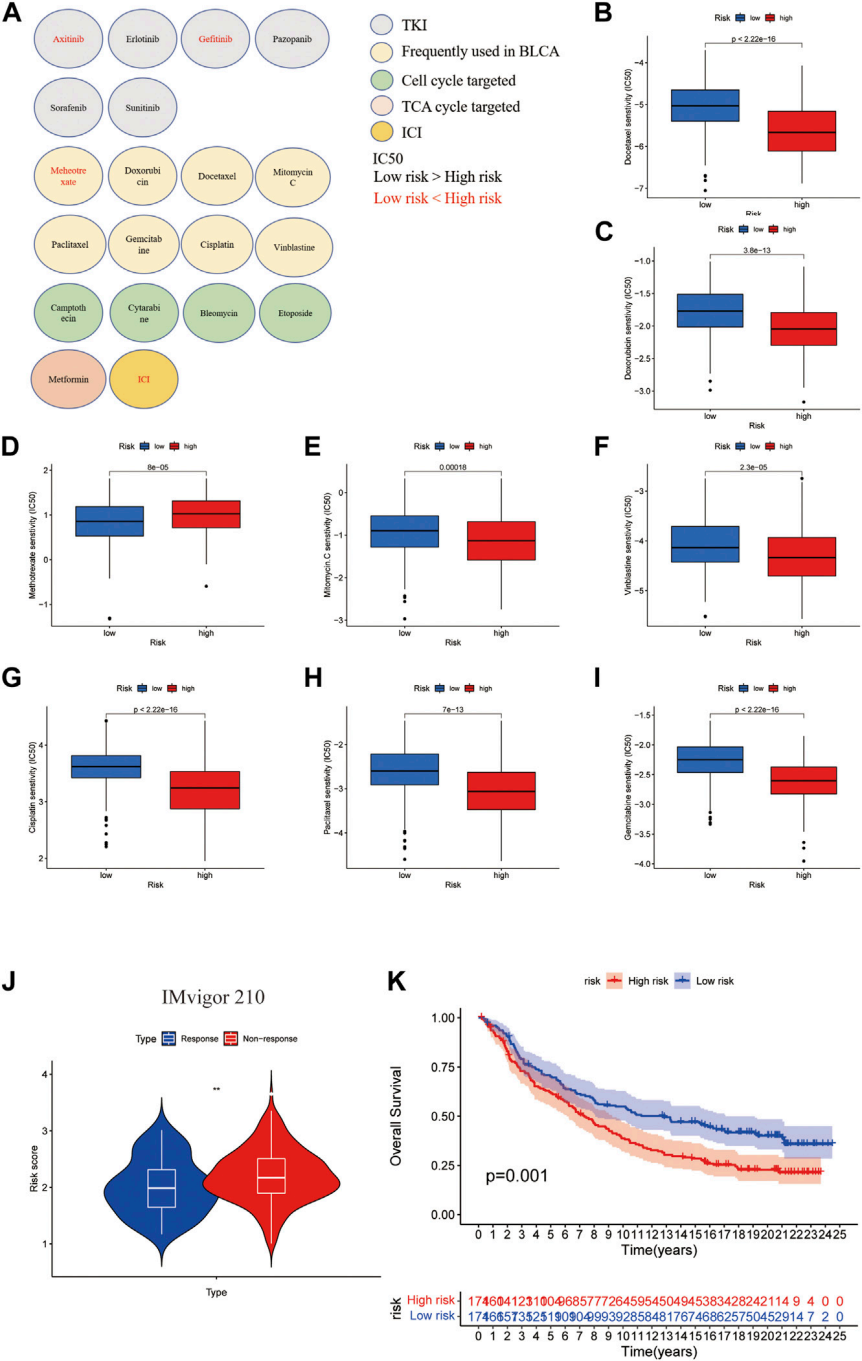
Drug sensitivity evaluation in cuproptosis risk groups. **(A)** An overview of roles of the cuproptosis scoring system on response to chemotherapy, targeted therapy, and immunotherapy. Drugs with lower IC50 in the low-risk group were shown in red. Drugs with lower IC50 in the high-risk group were shown in black. Except for ICI, the drug sensitivities were evaluated by the pRRophetic package. **(B–I)** Boxplots showed the drug sensitivities of chemotherapy drugs that are frequently used in BLCA treatment in high- and low-risk groups. **(J)** Boxplot showed the response of ICI evaluated in high- and low cuproptosis risk patients in the IMvigor210 cohort. **(K)** Kaplan-Meier curves for high and low cuproptosis risk group patients in IMvigor210 cohort. **p < 0.01. Abbreviations: IC50, half maximal inhibitory concentration; BLCA, bladder cancer; ICI, immune checkpoint inhibitor.

To give guidance for clinical treatment, we next compared the differences in the estimated relative half maximal inhibitory concentration (IC_50_) levels of several commonly used targeted-therapy drugs and chemotherapy drugs using pRRophetic package. As shown in Figure 9A, among the tyrosine kinase inhibitors (TKIs), the low-risk group tended to be more sensitive to Axitinib and Gefitinib. Although patients in the high-risk group had poor survival, they tended to be more sensitive to the chemotherapy drugs that are frequently used in BLCA except for Methotrexate. Since former GO and KEGG analysis showed that TCA cycle and cell cycle might be possible targets for patients who had high expression of CuGs, other cell cycle targeted drugs and TCA cycle targeted drugs were also evaluated, and proved to be more sensitive in high-risk group (Figures 9A–I). Since the tumor immune microenvironment showed a distinct difference between the two risk groups, the response of ICI was also predicted using the IMvigor210 cohort, a cohort of platinum-treated locally advanced or metastatic urothelial carcinoma (mUC) patients receiving anti-PD-L1 immunotherapy. Using the same grouping strategy, the high-risk group patients in the IMvigor210 cohort showed a significantly higher proportion of non-response (Figure 9J). The Kaplan-Meier curve indicated that patients in the high-risk group had significantly worse overall survival, which was an external validation of the cuproptosis scoring system (Figure 9K). These data proved that the cuproptosis scoring system could successfully estimate drug sensitivity and guide clinical practice.

### Detection of mRNA expression of the CuGs by qPCR

**FIGURE 10:**
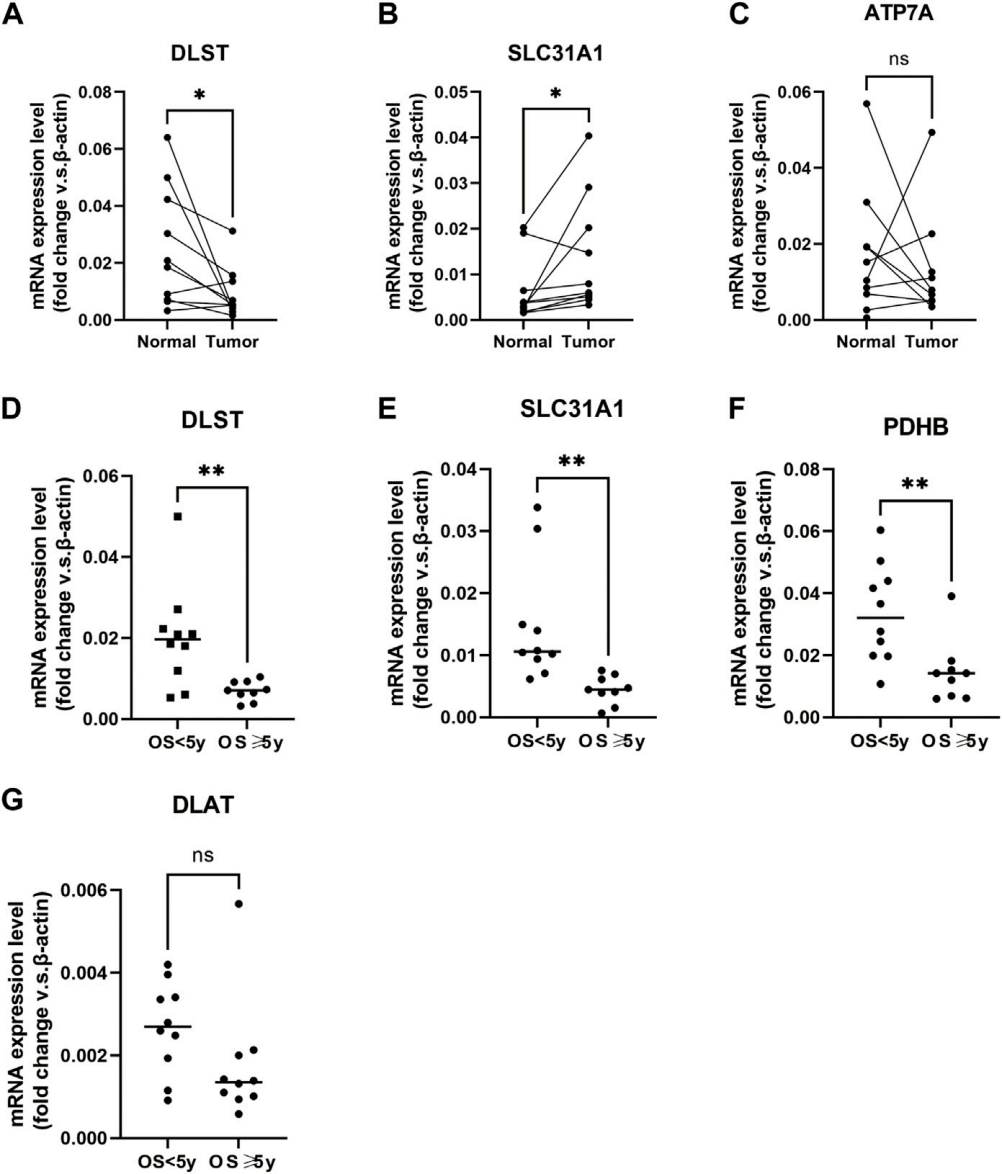
Verification of mRNA relative expression of the CuGs by qPCR **(A–C)** mRNA relative expression of DLST, SLC31A1, and ATP7A in 10 pairs of normal and tumor tissues of BLCA. **(D–G)** mRNA relative expression of DLST, SLC31A1, PDHB, and DLAT in 20 tumor tissues of BLCA. ns no significance, *p < 0.05, **p < 0.01. Abbreviations: CuGs, cuproptosis genes; qPCR, quantitative real-time PCR; BLCA, bladder cancer.

To further verify the results, we detected the mRNA relative expression of the differentially expressed CuGs in 10 pairs of normal and tumor tissues by qPCR. Consistently, the results showed that DLST and SLC31A1 were expressed differentially between normal tissues and tumor tissues (Figures 10A,B). While the expression of ATP7A did not show any difference (Figure 10C). We also detected the CuGs which were correlated with OS (*p* < 0.05 in univariate Cox regression) in another 20 tumor tissue from BLAC patients. As shown in Figures 10D–F, patients with an OS longer than 5 years had lower expression of DLST, SLC31A1, and PDHB compared with those who had an OS less than 5 years. Although the expression of DLAT did not show a significant difference, the trend can still be seen (Figure 10G). More samples may be required for further validation.

## Discussion

In this study, we revealed the expression profile, mutation frequency of the CuGs, and their correlation with OS in BLCA patients. Besides, we comprehensively analyzed the survival outcomes, signaling pathways, and TME features of different cuproptosis molecular patterns and cuproptosis gene clusters. Furthermore, the cuproptosis score system was established to assess the prognosis, tumor characteristics, TME feature, and drug sensitivity of every patient, which could help oncologists make more individualized treatment strategies.

In BLAC TCGA datasets, we could see DLST, encoding the essential component of the PDH complex, and ATP7A, encoding the important copper exporter showed a higher expression in normal tissues. While SLC31A1, which encodes the copper importer had a higher expression in tumor tissues. The results pointed out that copper is more likely to accumulate in tumor tissues and induce cuproptosis. However, except for LIPT1, all the CuGs were risk factors of OS in BLCA. Higher SLC31A1 was not correlated with better OS, which implied that the OS of patients could not be simply predicted depending on the expression of the single CuG. Further clusters are needed to predict the prognosis of patients more accurately.

Based on the mRNA expression profiles of 12 CuGs, we developed two cuproptosis molecular patterns for BLCA. Patients in cuproptosis pattern A had a significantly better overall survival than those in pattern B. There were significant differences in immune cell infiltration and immune statuses between the two patterns. Pattern B showed a more immunosuppressing TME. Function analysis of the differential genes between the cuproptosis molecular patterns revealed that pattern B showed higher activities on the TCA cycle and cell cycle, implying that inducing cuproptosis and targeting the cell cycle might be effective for these patients.

According to the DEGs between the two cuproptosis patterns, two gene clusters with unique prognostic and TME characteristics were constructed. Patients in cluster A who had lower CuGs expression observed a significant longer overall survival time than those in cluster B.

By using LASSO Cox regression, a cuproptosis scoring system was generated to calculate a specific cuproptosis score for every patient. Patients with high cuproptosis scores had higher CuGs expression and exhibited worse overall survival. ROCs proved its reliability for predicting the 1-, 3-, and 5-years survival rates of BLCA patients. After adjusting for other confounding factors, the cuproptosis score was confirmed to be an independent predictor for OS in BLCA patients. Since the cuproptosis score was correlated to the prognosis of BLCA, a nomogram was constructed combined with other clinicopathological characteristics to predict survival for every patient. ROCs showed that the prediction efficiency of the nomogram was better than using only traditional prediction markers, such as T stage or tumor grade.

TME has been increasingly accepted to play an integral and indispensable role in tumor anatomy and physiology. TME consists of stromal cells, immune cells, and the factors that they release around tumor cells (Cao et al., 2021). The relationship of cuproptosis with TME has not been studied yet. Our data revealed that a higher cuproptosis score was associated with immunosuppressing TME in BLCA, featured by higher infiltration levels of eosinophils, neutrophils, and M2, while lower infiltration levels of plasma cells, activated CD4 memory T cells, and CD8 cells. ESTIMATE algorithm showed that patients in the high cuproptosis score group had distinct higher stromal and immune scores than those in the low cuproptosis score group. Immune checkpoints also showed higher expression in the high cuproptosis score group. These indicated that CuGs could be correlated to the reconstruction of TME, hence influencing tumor growth and prognosis. Patients with high TMB and neoantigen burden tend to have better responses to immune therapies. Our data also revealed that patients with high cuproptosis scores had lower neoantigen load and TMB, which might be associated with their lower response to ICIs and worse overall survival.

An unbiased consensus clustering identified five MIBC molecular subtypes according to the mRNA expression profile based on the TCGA database. The molecular subgroup classes included luminal, luminal-infiltrated, basal-squamous, neuronal, and luminal-papillary, each having a distinct mutation profile and clinical outcomes (Robertson et al., 2017). So, we further probed into the mutation profile of patients with different cuproptosis scores to explore the relationship between the cuproptosis scoring system and TCGA molecular subtypes. Intriguingly, there were distinct mutation profiles in high and low cuproptosis score groups. In the low score group, the mutation profile was more similar to the luminal, luminal infiltrated, and luminal papillary subtypes, while in the high score group was more similar to the basal squamous subtype according to the 2017 TCGA clustering. To prove this, the risk score distribution of TCGA patients in five TCGA subtypes was applied according to the previous study. The results showed the consistency of the cuproptosis scoring system and the TCGA subtypes.

Since the TME was distinct, patients in different cuproptosis score groups might have different drug sensitivity. Despite the unfavorable survival outcome. Our data showed that patients with high cuproptosis scores tended to be more sensitive to the chemotherapy drugs that are frequently used in BLCA except for Methotrexate. Other cell cycle targeted drugs and TCA cycle targeted drugs could also be efficient in patients with high cuproptosis scores. A combination of these chemotherapy drugs and targeting cuproptosis might improve the prognosis of these patients. Response to ICIs was also evaluated. We found that patients with high cuproptosis scores showed significantly poorer response to ICIs treatments and worse overall survival. This indicated that the cuproptosis score could successfully predict drug sensitivity, thus helping oncologists make treatment decisions.

The prognostic values of the cuproptosis score have been validated in both internal and external datasets. The cuproptosis score system also worked in cohorts from our hospital. However, the current study still had some limitations. The prognosis prediction potency of the cuproptosis scoring system needed to be validated in a larger BLCA cohort in the real world. Although the drug sensitivities of frequently used chemotherapy drugs were evaluated, potential drugs such as cuproptosis targeted drugs and TCA cycle targeted drugs, which were not included in the pRRophetic package could not be assessed. The response of ICI was only evaluated in UC patients receiving PD-L1 immunotherapy. More validations in BLCA cohorts experiencing PD-1 immunotherapies are required.

## Conclusion

Taken together, this study depicted the landscape of cuproptosis in BLCA. We identified two cuproptosis molecular patterns and two cuproptosis gene clusters, with distinct survival outcomes, signaling pathways, and TME. We constructed a cuproptosis scoring system to predict the prognosis of BLCA patients. There were significant differences in TME, mutation profile, and drug sensitivities in high and low cuproptosis score patients. The cuproptosis scoring system could help oncologists comprehensively understand the tumor characteristic of BLCA and make individualized treatment strategies.

## Data Availability

The original contributions presented in the study are included in the article/Supplementary Material, further inquiries can be directed to the corresponding author.
