# Supplementary material for: Revealing prognostic and tumor microenvironment characteristics of cuproptosis in bladder cancer by genomic analysis
Source: Front Genet. 2022 Oct 3;13:997573. doi: 10.3389/fgene.2022.997573 (PMC9575963; doi:10.3389/fgene.2022.997573)
Supplement: Supplementary file 1 [file Table1.DOCX]

| **Gene Name** | **Forward Primer(5'to3')** | **Reverse Primer(5'to3')** |
| --- | --- | --- |
| **ATP7A** | TGACCCTAAACTACAGACTCCAA | CGCCGTAACAGTCAGAAACAA |
| **DLST** | GAACTGCCCTCTAGGGAGAC | AACCTTCCTGCTGTTAGGGTA |
| **SLC31A1** | GGGGATGAGCTATATGGACTCC | TCACCAAACCGGAAAACAGTAG |
| **PDHB** | AAGAGGCGCTTTCACTGGAC | ACTAACCTTGTATGCCCCATCA |
| **DLAT** | CGGAACTCCACGAGTGACC | CCCCGCCATACCCTGTAGT |
