# Supplementary figures and images for: Revealing prognostic and tumor microenvironment characteristics of cuproptosis in bladder cancer by genomic analysis
Source: Front Genet. 2022 Oct 3;13:997573. doi: 10.3389/fgene.2022.997573 (PMC9575963; doi:10.3389/fgene.2022.997573)

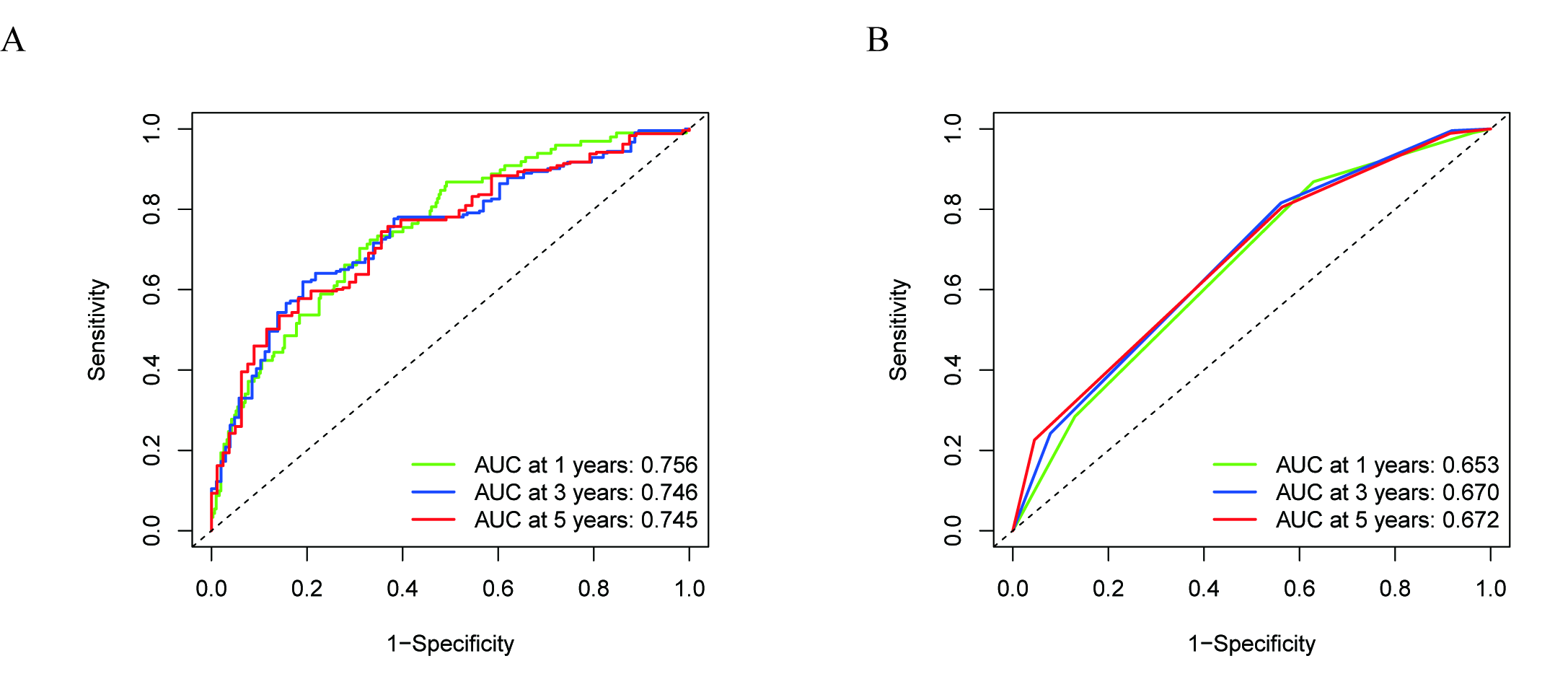

Supplement: Supplementary file 3 [file Image3.TIF]

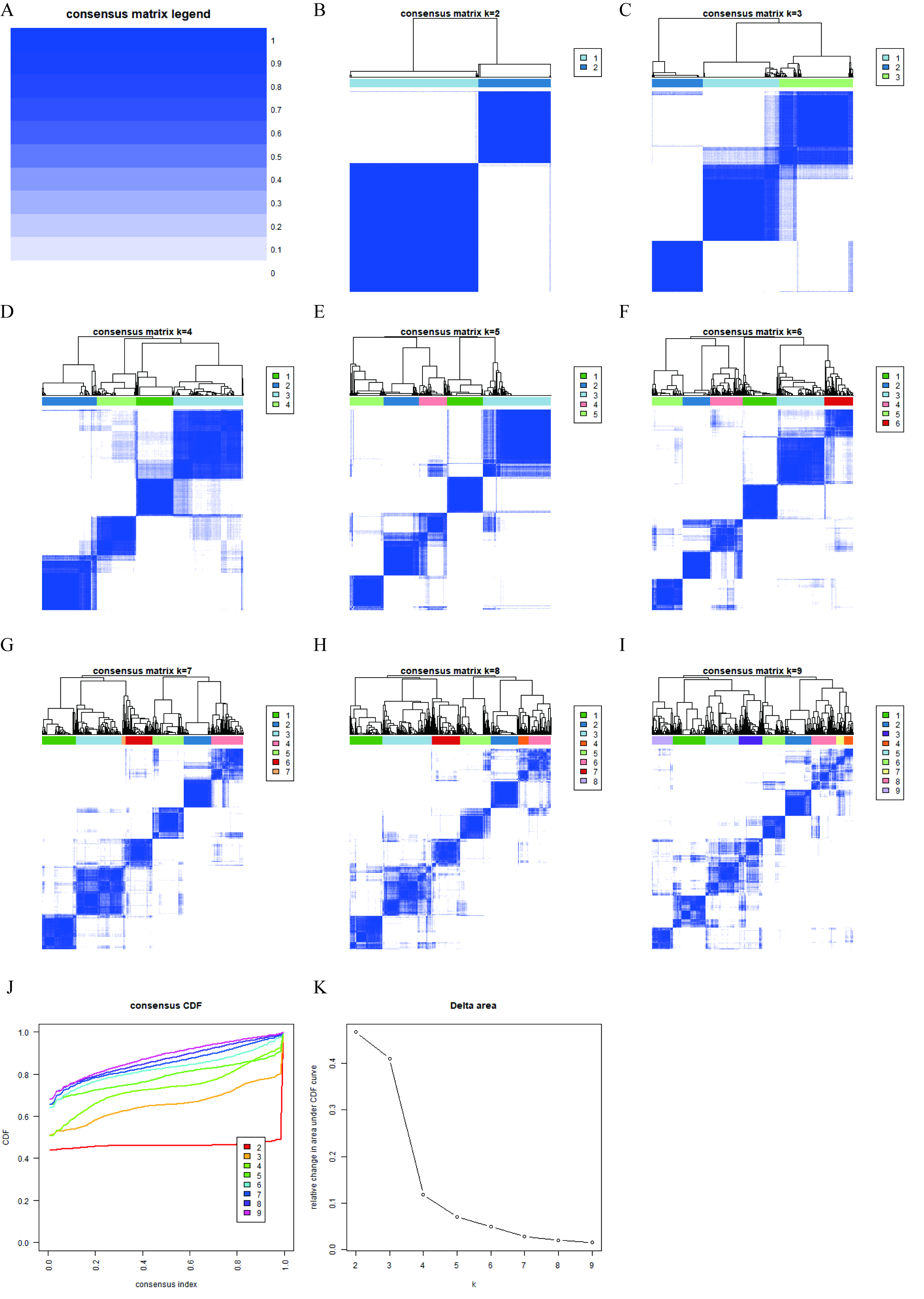

Supplement: Supplementary file 4 [file Image2.TIF]

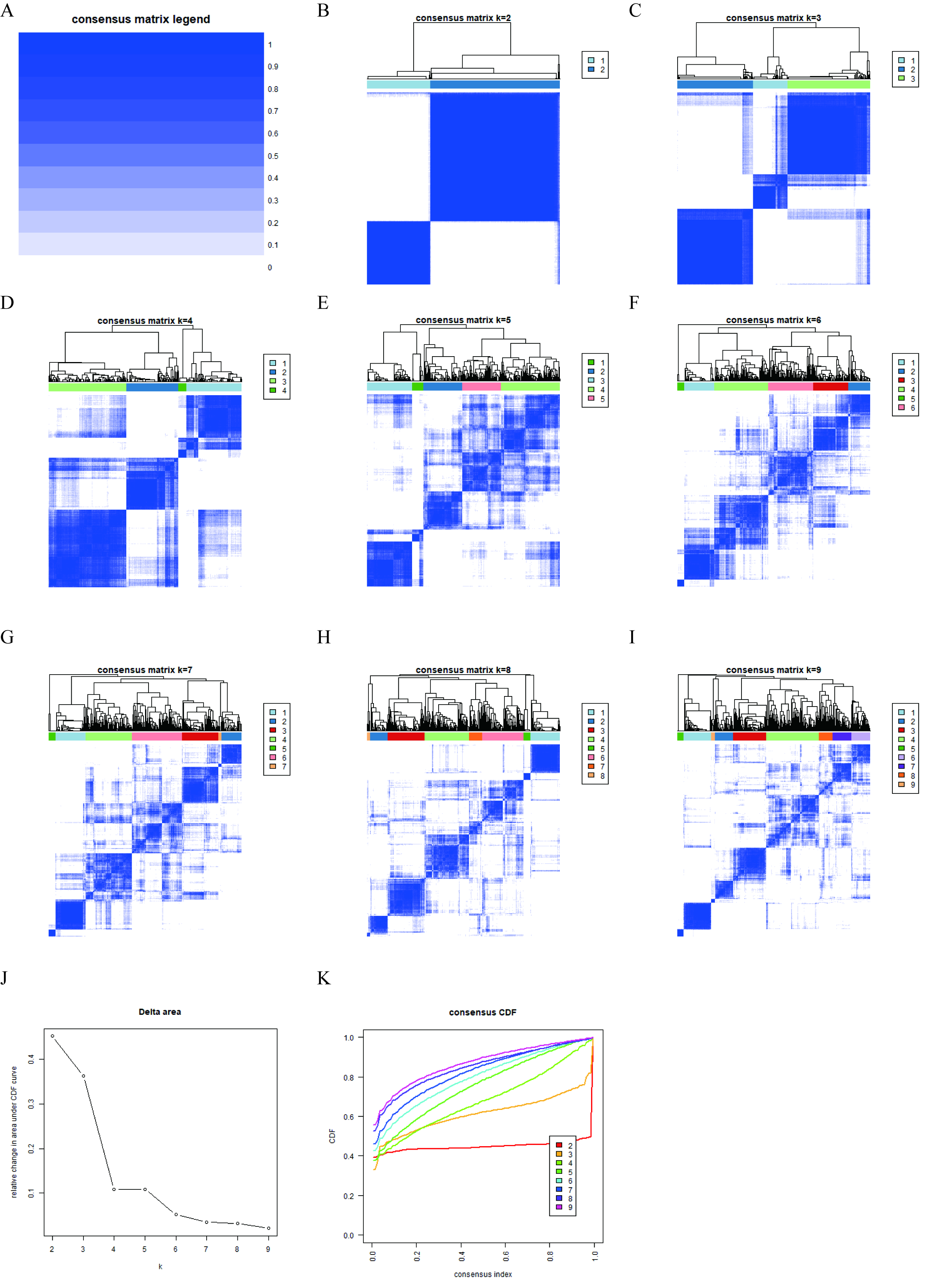

Supplement: Supplementary file 5 [file Image1.TIF]
